# Warning of the poor effect of chemotherapy: primary gastric malignant melanoma misdiagnosed as “gastric adenocarcinoma”: a case report and literature review

**DOI:** 10.3389/fonc.2026.1893937

**Published:** 2026-08-06

**Authors:** Yuting Zhang, Ruzhu Wang, Tianshu Yang, Jingwen Chen, Ping Gao, Ruiguo Zhao

**Affiliations:** 1 Department of Oncology, First Affiliated Hospital of Dalian Medical University, Dalian, Liaoning, China; 2 Institute of Integrated Traditional Chinese and Western Medicine, Dalian Medical University, Dalian, Liaoning, China

**Keywords:** backline treatment, case report, neoadjuvant chemotherapy, pathological misdiagnosis, primary gastric melanoma

## Abstract

Primary gastric melanoma is a rare clinical finding. Its diagnosis can be challenging due to the complex tissue structure of the stomach and the diverse morphology of melanoma cells, which contribute to the increased likelihood of misdiagnosis. Here we report a 69-year-old female patient with primary gastric melanoma, initially misdiagnosed as “gastric adenocarcinoma”. The patient initially received two cycles of neoadjuvant therapy with Tegeo in combination with oxaliplatin (SOX). Unfortunately, the response was not satisfactory. In order to guide further treatment decisions, immunohistochemistry (IHC) and next-generation sequencing (NGS) were conducted. Immunohistochemistry: CK (-), CK8/18 (-), Vimentin (+), CD56 (-), CgA (-), Syn (-), ALK (-), CD30 (-), S-100 (+), INI1 (expression), CD43 (-), CD3 (-), CD20 (-), Brg-1 (expression), CD117 (-), DOG-1 (-), CD34 (-), HMB-45 (+), melan-A (+), Her-2 (-). NGS showed no significant abnormalities in HER-2 and wild type in the range of BRAF gene detection. Through a Multidisciplinary Team (MDT) consultation, she was finally diagnosed with “primary gastric malignant melanoma”. Despite the disease progressing after first-line chemotherapy with temozolomide, cisplatin, and bevacizumab, a second-line treatment with albumin paclitaxel, carboplatin, and bevacizumab resulted in a sustained partial remission (PR). The patient’s condition was stable during the follow-up period. As far as we know, this is a rare case of primary gastric malignant melanoma which received persistent PR after second-line treatment. Combined with the pathological manifestations of MM and recent clinical studies, the pathological features and treatment of primary gastric malignant melanoma were discussed.

## Introduction

Extradermal melanoma (Extracutaneousmelanoma, ECM) is a clinical rarity. Primary gastric melanoma is extremely rare, accounting for only 1% of all extracellular melanomas. It mostly occurs in the elderly population, with no gender difference, and is often diagnosed in advanced stages with very poor prognosis (1). The tissue structure of gastric melanoma is complex and the cell morphology is diverse. Typically, different cell morphologies and growth patterns are intermixed in the same case of malignant melanoma. Misdiagnosis can easily occur, especially when there is no melanosis in the tumor tissue. Neoadjuvant therapy is currently the standard of care for locally advanced gastric cancer, but in the field of melanoma, the neoadjuvant approach remains controversial and has not yet been included in treatment guidelines as a recommended treatment with class IA evidence (2). Currently, the standard management of localized and oligometastatic melanoma is systemic adjuvant therapy after surgery. For the completely resectable locally advanced population, surgery remains the treatment of choice. However, 10-15% of stage III melanoma patients cannot undergo resection because of the tumor size, tumor location, or expected surgical complications. For these patients, neoadjuvant systemic therapy can be considered as a potential treatment strategy. The combination therapy in neoadjuvant therapy of primary gastric melanoma is still controversial, and there is no systematic study to date.

The highlight of our case is that when the effect of neoadjuvant chemotherapy is limited, it warns us to reconsider the pathological diagnosis through MDT consultation. The utilization of a combination of chemotherapy and anti-angiogenesis in the treatment of primary gastric melanoma (MM) in our case is relatively uncommon when compared to the standard neoadjuvant chemotherapy regimen of temozolomide combined with cisplatin. This unique approach may offer valuable insights to clinicians and serve as a foundation for future clinical trials aiming to explore novel treatment options for primary gastric melanoma.

## Case presentation

**Figure 1:**
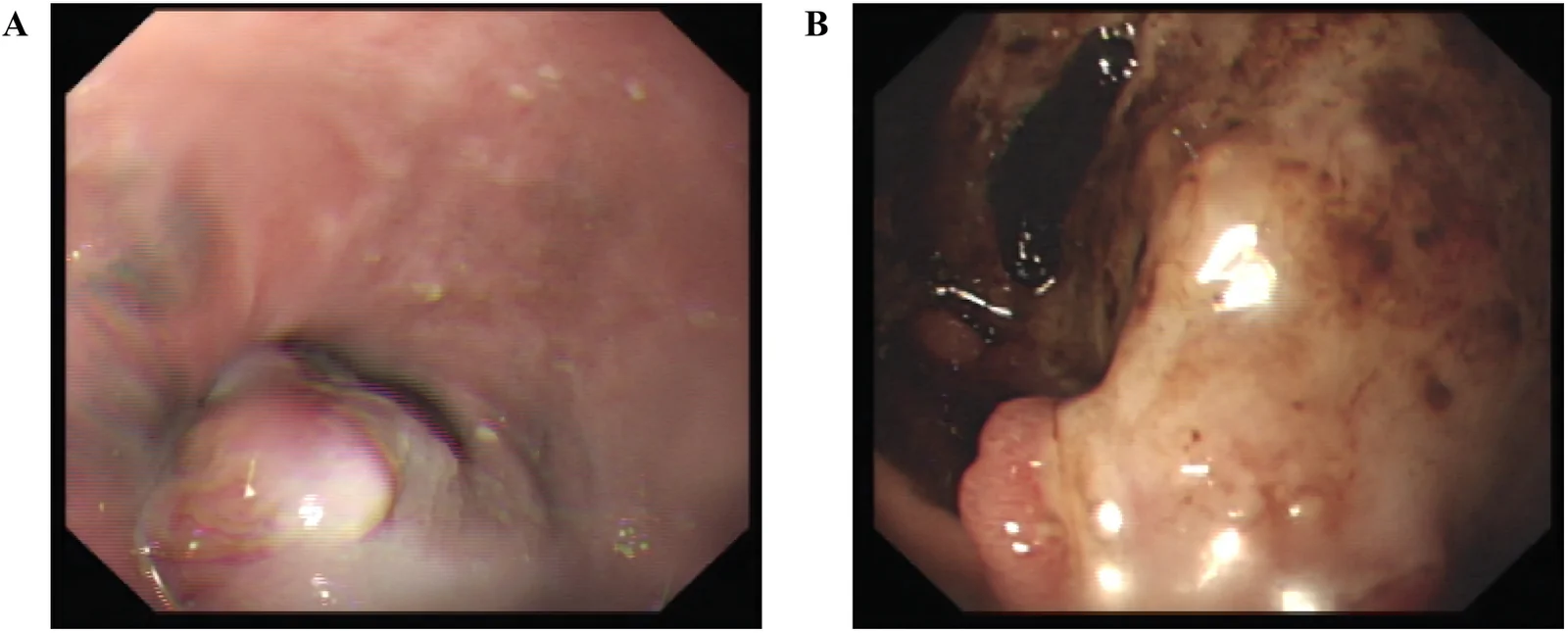
Gastroscopic manifestations. **(A)** Initial endoscopic view of the lesion; **(B)** Magnified view of the irregular ulcerative lesion. The gastroscopic findings of the patient showed irregular ulcerative lesions in the annular cavity between the cardia and gastric fundus.

On November 16, 2022, a 69-year-old woman was admitted to a local hospital, presented with dysphagia for seven days accompanied by fatigue and progressive weight loss. The patient had no previous history of other diseases or complications, no alcohol and tobacco dependence, and no known family or hereditary diseases. A physical examination showed upper abdominal tension and tenderness. Gastroscopy showed that the ring cavity between the lower end of the esophagus, cardia, and fundus of the stomach was an irregular ulcerative lesion (**Figure 1**), and pathology showed “adenocarcinoma”.

**Figure 2:**
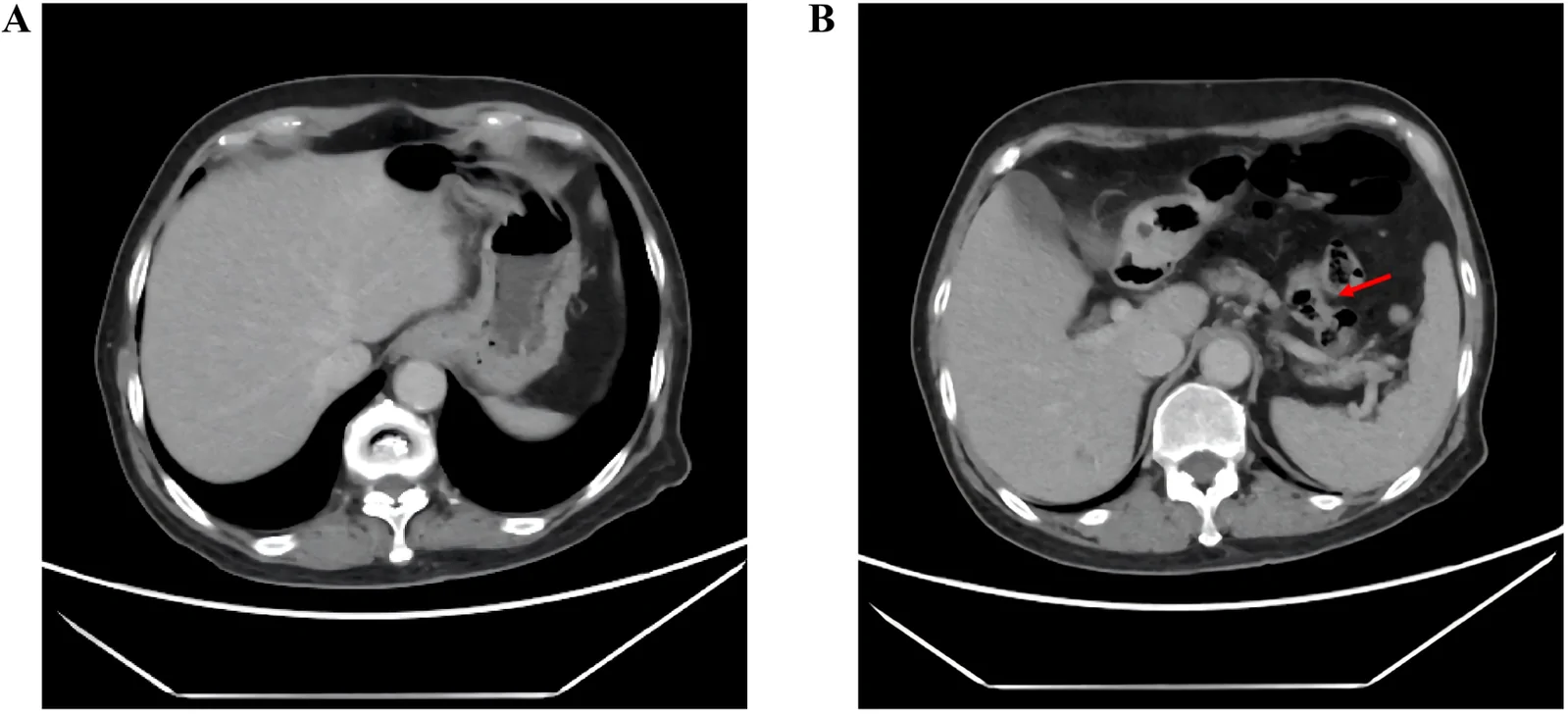
The enhanced abdominal CT showed: **(A)** The wall of the lower end of the esophagus, cardia, and stomach is thick, with a diameter of about 4.32cm. **(B)** Multiple enlarged lymph nodes were seen around the stomach, and the short diameter of the large ones was about 1.2cm.

Without any treatment, the patient was then transferred to our hospital for further examination. The enhanced abdominal CT showed the wall thickness of the lower end of the esophagus, cardia, and stomach (**Figure 2A**). Considering the malignant tumor, multiple enlarged lymph nodes could be seen around the stomach, partially fused, and the boundary with the adjacent gastric wall was not clear (**Figure 2B**). The serum level of carcinoembryonic antigen (CEA) was 25ng/mL(normal reference: <5 ng/mL for non-smokers, <10 ng/mL for smokers), and sugar antigen (CA724) was 19.4kU/L(normal reference: <6.9 kU/L). There were no obvious signs of metastasis in lung CT and head CT. The above imaging examination combined with pathology confirmed that the patient was diagnosed as cT3N+M0, stage III gastric adenocarcinoma.

**Figure 3:**
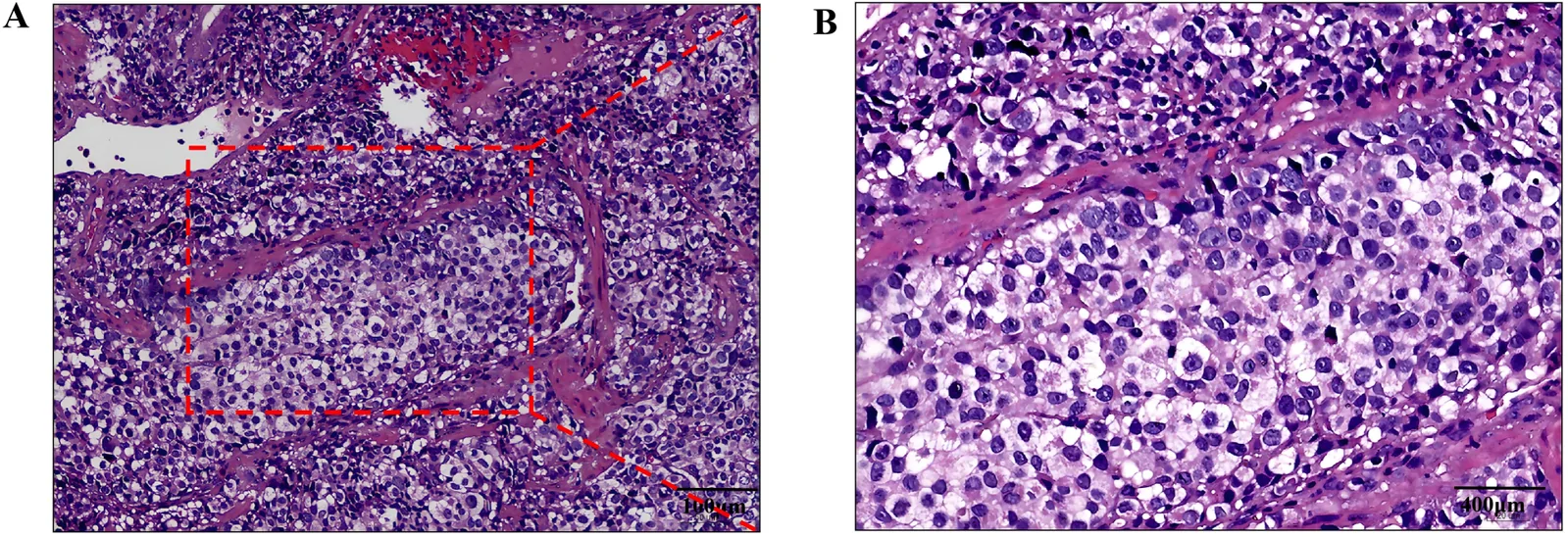
Microscopic morphology: **(A)** HE×100; **(B)** HE×400.

**Figure 4:**
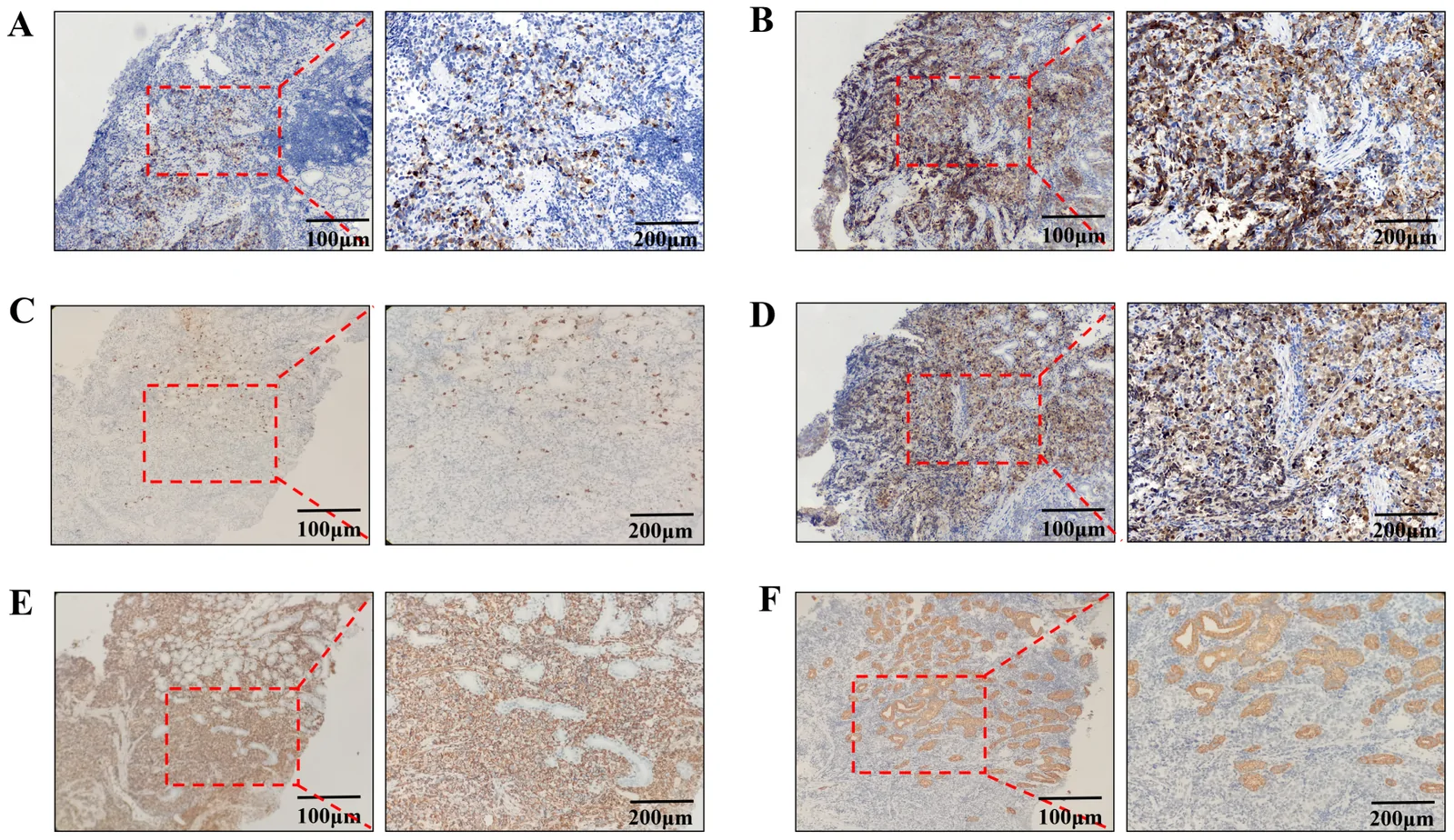
Results of immunohistochemical staining (EnVision™ Detection System, ×200). **(A)** HMB45 (+) (clone HMB-45, ready-to-use); **(B)** Melan (+) (clone MX118, ready-to-use); **(C)** CgA (-) (clone MX018, ready-to-use); **(D)** S100 (+) (clone rabbit polyclonal, ready-to-use); **(E)** Vimentin (+) (clone MX034, ready-to-use); **(F)** Ck (-) (clone AE1/AE3,ready-to-use);.

Our patient presented with increased dysphagia after 2 cycles of chemotherapy with the SOX regimen. The enhanced abdominal CT showed increased thickness of the lower end of the esophagus, cardia, gastric base compared to previous scans, while theperigastric lymph nodes appeared to remain the same. In order to improve therapeutic efficacy, IHC and NGS were performed. Next-generation sequencing (NGS) was performed on archival tumor tissue using a targeted sequencing panel (Tumor Hotspot Gene Standard Detection – Melanoma, 2021 version) on the Illumina platform (Novaseq). The panel covered 84 genes relevant to melanoma, including but not limited to BRAF, KIT, NRAS, KRAS, TP53, NF1, GNAQ, GNA11, PTEN, AKT1, MAP2K1, NTRK1/2/3, ALK, ROS1, RET, MET, PIK3CA, EGFR, ERBB2, CTNNB1, APC, TCF7L2, and mismatch repair genes (MSH2, MLH1, MSH6, PMS2). Microsatellite instability (MSI) status was evaluated as part of the assay; however, tumor mutational burden (TMB) was not calculated by this specific panel.IHC analysis indicated that the tumor cells were (**Figure 3**): CK (-), CK8/18 (-), Vimentin (+), CD56 (-), CgA (-), Syn (-), ALK (-), CD30 (-), S-100 (+), INI1 (expression), CD43 (-), CD3 (-), CD20 (-), Brg-1 (expression), CD117 (-), DOG-1 (-), CD34 (-), HMB-45 (+), melan-A (+), Her-2 (-). *In situ* hybridization for EBER was also negative(**Figure 4**). The NGS assay revealed wild-type sequences for BRAF (including V600E and non-V600E loci), KIT (exons 11 and 17), NRAS, and KRAS, with no detectable pathogenic mutations in TP53. The microsatellite status was classified as microsatellite stable (MSS). Additional variants of uncertain significance were identified, including a splice-site alteration in TCF7L2 (c.484-2A>T, variant allele frequency 59.03%) and a missense mutation in NTRK1(c.1387A>G p.Met463Val,16.82%).No fusion events involving ALK, ROS1, NTRK1/2/3, or RET were detected.The patient was examined by MDT consultation of dermatology, ophthalmology, stomatology, and anorectal surgery, and no tumor was found to exist in other parts of the body. Combined with the pathology and immunohistochemistry results, the patient was considered to be a primary gastric melanoma, but the patient refused to undergo a PET-CT examination due to financial constraints., and we were unable to make a further definitive diagnosis. The patient was finally diagnosed with primary gastric mucosal melanoma, which was locally advanced and could not be completely resected.

## Therapeutic interventions and follow-up

**Figure 5:**
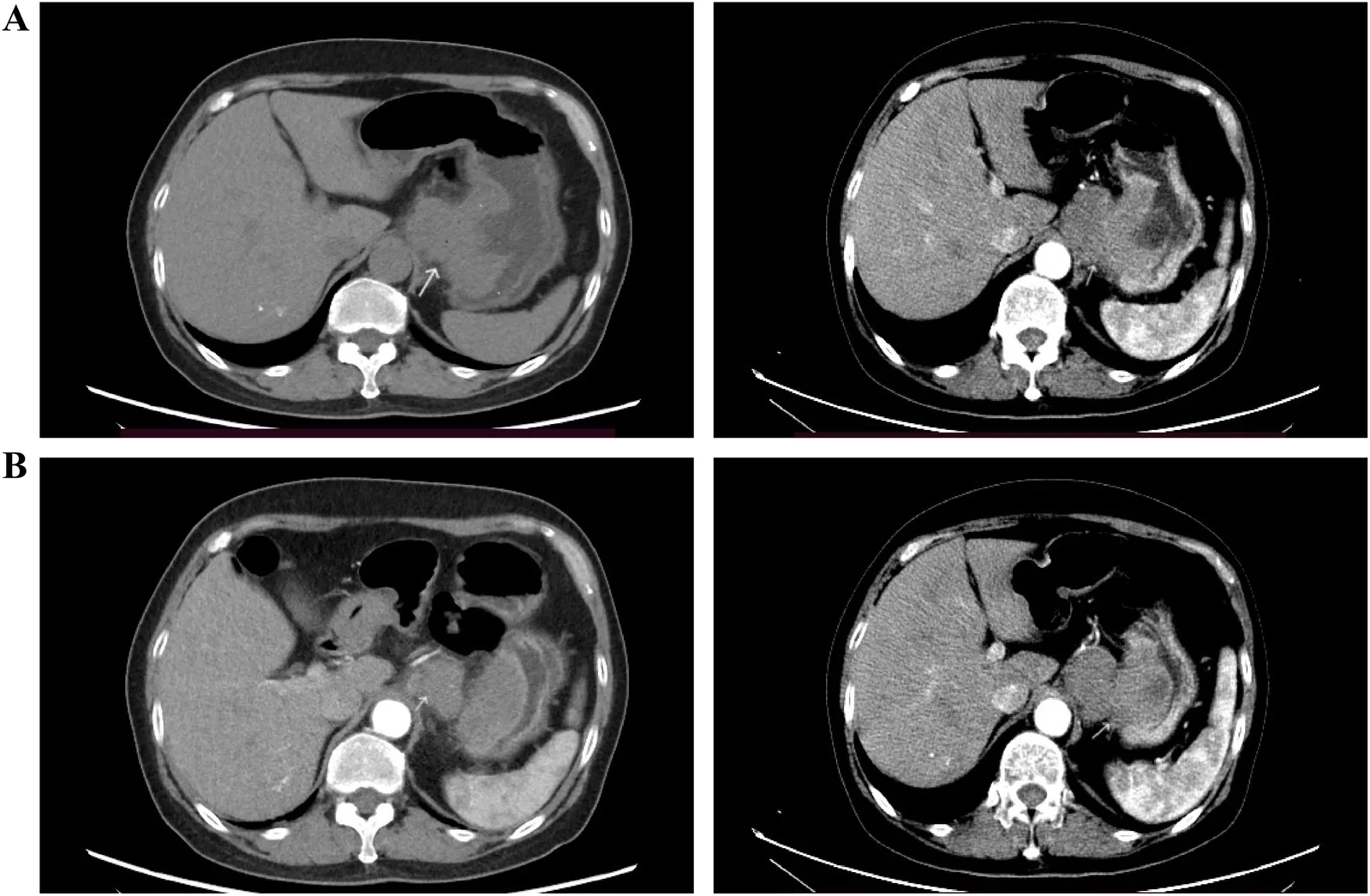
Computed tomography (CT) showed: **(A)** 2022.12 (right) and 2023.01 (left), and the wall thickness of the lower esophagus, cardia, and stomach was about 5.42cm, which was significantly larger than that of the anterior (4.32cm). **(B)** 2022.12 (right) and 2023.01 (left), and there were multiple enlarged lymph nodes around the stomach, with a short diameter of about 1.5cm, larger than that of the anterior (1.2cm).

**Figure 6:**
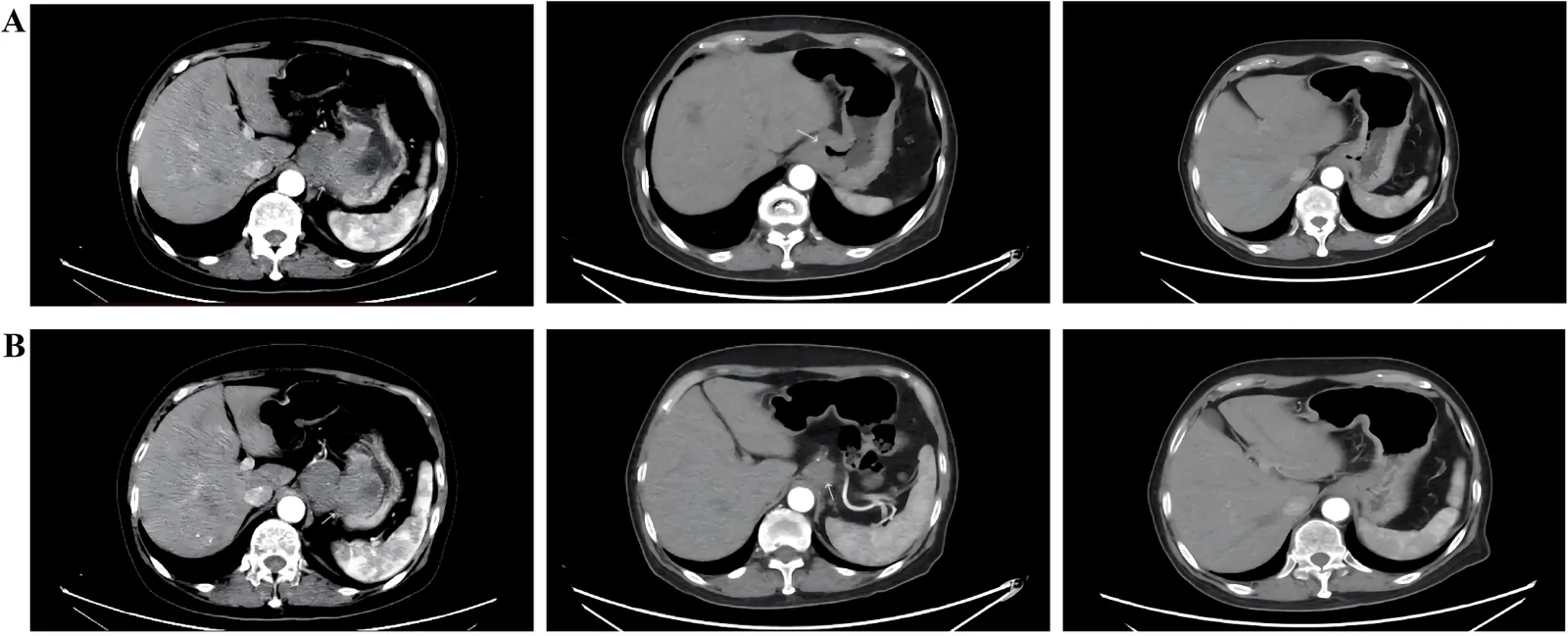
Computed tomography (CT) showed from the right to the left: Jan.2023, Apr. 2023, Jun.2023. **(A)** The wall thickness of the cardia and the bottom of the stomach were seen as follows: 5.42cm→3.84cm→2.54cm, it is significantly smaller than before. **(B)** There were many enlarged lymph nodes around the stomach, and the short diameter of the large ones almost disappeared by 1.5cm”.

From December 5, 2022, to January 16, 2023, the patient received neoadjuvant chemotherapy with the SOX regimen (S-1 plus oxaliplatin) for 2 cycles. Restaging contrast-enhanced CT performed on March 10, 2023, demonstrated disease progression, with increased wall thickness at the lower esophagus, cardia, and gastric fundus (from 4.32 cm to 5.42 cm), as well as enlarged perigastric lymph nodes (short diameter from 1.2 cm to 1.5 cm) (**Figure 5**). Given the poor response to neoadjuvant therapy and the revised pathological diagnosis, the treatment regimen was switched to first-line therapy in late March 2023, consisting of temozolomide (100 mg, once daily, days 1–3) plus cisplatin (80 mg/m², days 1–2) combined with bevacizumab (15 mg/m², day 1) for 2 cycles (3 weeks per cycle). However, this first-line regimen also failed to control the disease, with confirmatory imaging showing further progression. Consequently, beginning in April 2023, the patient received second-line therapy, consisting of albumin-bound paclitaxel (260 mg/m², once daily, day 1) plus carboplatin (AUC = 5) combined with bevacizumab (15 mg/m², once daily). After 2 cycles, abdominal CT showed a partial remission (PR) of the primary tumor and lymph nodes according to RECIST v1.1 (**Figure 6**). The patient completed six cycles of this therapy and achieved a sustained PR, without treatment-related adverse events such as myelosuppression or bleeding. However, the patient refused surgical intervention and opted to continue the original regimen as maintenance therapy. After the initial progression documented in March 2023, the disease remained stable by November 2023; nevertheless, the patient declined further treatment options including surgery and alternative systemic therapies, after thorough discussion with the medical team. A repeat CT scan in February 2024 demonstrated continued tumor shrinkage compared with the previous evaluation. Nonetheless, the patient persisted in refusing further intervention. Unfortunately, she eventually succumbed to disease progression in June 2024. The timeline of the entire clinical course is summarized in **Table 1**.

**Table 1:** The clinical course of disease over time. It includes treatment, diagnostic procedures, and the timing of disease progression.

Date	Treatment / event	Response	Note
2022-11-16	Admitted to hospital; gastroscopy and biopsy; initial pathological diagnosis: "gastric adenocarcinoma"	—	Baseline staging: cT3N+M0, stage III
2022-12-05 to 2023-01-16	Neoadjuvant chemotherapy (SOX regimen): S-1 + oxaliplatin, 2 cycles	—	Initial treatment per gastric cancer guidelines
2023-03-10	Restaging contrast-enhanced CT: primary tumor thickened (5.42 cm), lymph nodes enlarged (1.5 cm)	**PD (progressive disease)**	SOX regimen ineffective; prompted diagnostic re-evaluation
2023-03-15	Immunohistochemistry (IHC) and next-generation sequencing (NGS); MDT consultation	—	Final diagnosis revised to **primary gastric malignant melanoma** (BRAF/KIT/NRAS wild-type, MSS)
2023-03-20 to mid-April 2023	First-line therapy: temozolomide + cisplatin + bevacizumab, 2 cycles	**PD** (confirmed on subsequent evaluation)	First-line ineffective; disease continued to progress
2023-04-01 to 2023-10-15	Second-line therapy: nab-paclitaxel + carboplatin + bevacizumab, 6 cycles	**PR (partial remission)** (achieved after 2 cycles, sustained through 6 cycles)	Patient refused surgery; continued same regimen as maintenance
2023-11-30	Maintenance therapy (same second-line regimen); follow-up CT	**SD (stable disease)**	Still maintained PR; patient refused further treatment options
2024-02-15	Follow-up CT: continued tumor shrinkage compared with previous scan	**SD with further shrinkage**	Patient persisted in refusing surgery or change of therapy
2024-06-24	Death	—	Disease control duration from start of second-line: **>16 months**

All bold values in **Table 1** are quantitative imaging indicators measured on serial contrast-enhanced abdominal CT scans, including the maximum thickness of primary gastric malignant melanoma lesion and the maximal short-axis diameter of metastatic perigastric lymph nodes at each follow-up time point. These measurements are used to evaluate therapeutic response and disease progression following RECIST version 1.1 criteria. The table records the full clinical course of this unresectable locally advanced primary gastric mucosal malignant melanoma, documenting all treatment cycles, efficacy assessments, and an overall disease control survival of more than 16 months from the start of second-line chemotherapy to patient death due to tumor progression.

## Discussion

Malignant melanoma is an exceptionally aggressive type of cancer that progresses rapidly and often metastasizes to various organs. It exhibits a high level of malignancy and carries a bleak prognosis, with a 20% rate of metastasis. MM usually account for less than 2% of all melanoma types, but their prognosis is usually poor (3). Mucosal melanoma is an extremely rare type of melanoma, mainly in the head and neck. Primary tumors are most often located in the nasal cavity, paranasal sinus, and oral cavity, and mucosal melanoma in the stomach is very rare (4). Malignant melanoma poses significant challenges in terms of diagnosis due to its distinct clinical and histological presentations, making it prone to misdiagnosis. Apart from the intricate histomorphological variations, an endoscopic biopsy can also contribute to potential “misdiagnosis.” This is because an endoscopic biopsy can only capture one or a few aspects, making it challenging to observe the characteristic manifestations of malignant melanoma. On the other hand, surgical resection plane, lesion location, biopsy depth, and instrument quality will also affect the accuracy of pathological results (7).We note that although the histological morphology of malignant melanoma is complex and diverse, most tissue types typically exhibit HMB-45 (+), melan-A (+), Vimentin (+), S-100 (+), however, it is important to recognize that certain melanomas are notably devoid of S100 protein expression, and some may indeed show the presence of CKs, such as CK8/18, contrary to the classical immunophenotype (5, 12).A recent case report by Ren et al. documented an S100-negative primary anorectal melanoma with elevated Ki67, highlighting the diagnostic challenges posed by atypical immunophenotypes (5). Similarly, Saliba and Bhawan reviewed aberrant expression of immunohistochemical markers in malignant melanoma, emphasizing that cytokeratin expression, though unusual, can occur in a subset of melanomas and may lead to diagnostic confusion (12). In our case, the tumor cells were CK (-) and CK8/18 (-), which, combined with positive HMB-45, melan-A, S-100, and Vimentin staining, supported the diagnosis of melanoma. “Therefore, immunohistochemistry plays an important role in the diagnosis of melanoma without melanin granules. The molecular findings from NGS merit further discussion in the context of this case. The assay confirmed a microsatellite stable (MSS) phenotype and wild-type status across BRAF (including V600E), KIT, NRAS, KRAS, and TP53. The absence of actionable driver mutations is consistent with the genomic profile typically observed in mucosal melanoma, which generally harbors a lower tumor mutational burden compared with cutaneous melanoma. This may partly explain the limited efficacy often seen with single-agent immunotherapy in this subtype. The wild-type KIT result excluded the possibility of a gastrointestinal stromal tumor and ruled out the use of KIT-targeted tyrosine kinase inhibitors. Similarly, the absence of BRAF and NRAS mutations precluded the use of BRAF/MEK inhibitors, which are standard in cutaneous melanoma but have limited data in mucosal subtypes. Multifactorial analysis of studies has shown that BRAF gene mutations are an independent prognostic factor for melanoma (6), and our patient’s BRAF wild-type status, while precluding targeted therapy, may suggest a distinct prognostic subgroup, although its specific impact in mucosal melanoma remains to be fully elucidated. Collectively, these NGS data not only assisted in refining the differential diagnosis but also reinforced our therapeutic strategy of combining chemotherapy and antiangiogenic agents, which ultimately achieved a sustained partial remission.The tumor cells of this patient showed an epithelioid type, nesting arrangement, and obvious atypia, so it was misdiagnosed as “gastric adenocarcinoma” by the local hospital. IHC in our hospital leading to a correct diagnosis. Nevertheless, the initial biopsy was performed at a local hospital, and the corresponding paraffin blocks were not requested for review at our center prior to treatment initiation. Ideally, a review of the original biopsy material at the treating institution would have enhanced the diagnostic precision; however, this was not done in our case, which constitutes a limitation. Despite this, we subsequently performed comprehensive IHC and NGS on the available tissue to establish the correct diagnosis.

Several studies have confirmed that complete surgical resection of gastrointestinal MM can not only control symptoms but also improve the prognosis, thus increasing the overall survival time (7). However, in certain clinical scenarios, surgical resection may not be feasible for some patients. According to the recommendations of CSCO (Chinese Society of Clinical Oncology)guidelines, the neoadjuvant therapy regimen of Tigio combined with oxaliplatin (SOX) was determined.

Studies of perioperative treatment (neoadjuvant radiotherapy + surgery + adjuvant radiotherapy/chemotherapy) for gastric cancer have confirmed that this treatment modality results in downstaging of the tumor, increased R0 resection rate and improved overall survival compared to surgery alone, without increasing postoperative complications and morbidity and mortality (8). For patients with poor neoadjuvant efficacy, further genetic testing can be performed, including HER2, CPS(Combined Positive Score), EBV(Epstein-Barr Virus), MMR(Mismatch Repair), etc, to guide the clinical use of drugs and improve the efficacy. In such cases, neoadjuvant systemic therapy can emerge as a promising treatment strategy. In the field of neoadjuvant therapy of MM, numerous clinical trials indicated that chemotherapy combined with antiangiogenic therapycan provide significant benefits to patients (9). Smith et al. conducted a comparative analysis between patients who received surgical resection either before and after effective systemic treatment (EST). However, it is important to note that their study included only 4 cases of mucosal melanoma, which substantially limits the generalizability of their findings. Among the subgroups analyzed, they observed that patients who underwent intraperitoneal resection of metastases exhibited the most notable increase in the proportion of surgeries performed following EST. Nevertheless, given the very small sample size of this specific subgroup, this observation should be interpreted with caution and requires validation in larger cohorts.A Chinese retrospective study presented at the 2018 ESMO conference showed that the first-line regimen (DTIC + cisplatin + endostar) achieved a median PFS of 4 months, and the second-line (paclitaxel + carboplatin + bevacizumab) achieved a median PFS of 2 months. A recent meta-analysis (10) further demonstrated that the addition of bevacizumab to chemotherapy significantly improved overall survival in patients with unresectable or advanced melanoma (HR = 0.64, 95% CI 0.49–0.85, p < 0.01). Taken together, these findings suggest that chemotherapy combined with antiangiogenic agents may be considered an alternative treatment option for this population. The effectiveness of bevacizumab combined with chemotherapy has been fully confirmed in the treatment of metastatic colorectal cancer (mCRC), non-small cell lung cancer (NSCLC), and breast cancer. However, its role in neoadjuvant therapy of MM is still uncertain. This patient with locally advanced primary gastric melanoma experienced continuous PR and surgical opportunity after posterior line therapy, which is much better than the results of most clinical studies of locally advanced MM. In this case, the patient exhibited a positive response to the combination treatment, demonstrating ongoing disease control. The additional survival time observed further underscores the superiority of this treatment approach in managing the disease in this specific instance.

## Conclusion

This case highlights that primary gastric melanoma, though rare, can be easily misdiagnosed as gastric adenocarcinoma due to its overlapping histological features. A high index of suspicion, combined with immunohistochemical staining (HMB-45, Melan-A, S-100) and multidisciplinary consultation, is essential for accurate diagnosis. Importantly, our patient showed a poor response to standard SOX neoadjuvant chemotherapy, which initially prompted a diagnostic re-evaluation and ultimately led to the correct diagnosis. After confirming primary gastric melanoma, first-line temozolomide/cisplatin/bevacizumab failed to control the disease, but a subsequent switch to albumin-bound paclitaxel, carboplatin, and bevacizumab achieved a sustained partial remission, but a subsequent switch to albumin-bound paclitaxel, carboplatin, and bevacizumab achieved a sustained partial remission, with disease control lasting for over 16 months until the patient’s eventual death due to disease progression.This experience suggests that, for locally advanced unresectable gastric melanoma, chemotherapy combined with antiangiogenic agents—particularly the paclitaxel/carboplatin/bevacizumab regimen—may offer a viable salvage option when conventional regimens are ineffective. However, the diagnostic challenge of not reviewing the original biopsy blocks from the referring hospital remains a limitation of this report, emphasizing the need for routine pathological review at the treating center (11).

## Data Availability

The original contributions presented in the study are included in the article/**Supplementary Material**. Further inquiries can be directed to the corresponding authors.

## Supplementary material

Supplementary Figure 1 Contrast-enhanced abdominal CT findings in February 2024.
